# Heuristics for Two Depot Heterogeneous Unmanned Vehicle Path Planning to Minimize Maximum Travel Cost

**DOI:** 10.3390/s19112461

**Published:** 2019-05-29

**Authors:** Jungyun Bae, Woojin Chung

**Affiliations:** Department of Mechanical Engineering, Korea University, Seoul 02841, Korea; jungyun.bae@gmail.com

**Keywords:** Multi-Robot Task Allocation, min-max Traveling Salesman Problem, Path Planning, Primal-dual heuristic

## Abstract

A solution to the multiple depot heterogeneous traveling salesman problem with a min-max objective is in great demand with many potential applications of unmanned vehicles, as it is highly related to a reduction in the job completion time. As an initial idea for solving the min-max multiple depot heterogeneous traveling salesman problem, new heuristics for path planning problem of two heterogeneous unmanned vehicles are proposed in this article. Specifically, a task allocation and routing problem of two (structurally) heterogeneous unmanned vehicles that are located in distinctive depots and a set of targets to visit is considered. The unmanned vehicles, being heterogeneous, have different travel costs that are determined by their motion constraints. The objective is to find a tour for each vehicle such that each target location is visited at least once by one of the vehicles while the maximum travel cost is minimized. Two heuristics based on a primal-dual technique are proposed to solve the cases where the travel costs are symmetric and asymmetric. The computational results of the implementation have shown that the proposed algorithms produce feasible solutions of good quality within relatively short computation times.

## 1. Introduction

In most applications of monitoring of large environments, the utilization of multiple autonomous unmanned vehicles (UVs) is preferred to improve system efficiency. For example, unmanned aerial vehicles (UAVs) are widely used in crop management [1,2], traffic monitoring [3], and forest management [4,5]. Various types of multiple UVs are widely used in surveillance [6,7,8], search and rescue applications [9,10,11], and transportation [12]. In most of these applications, the fundamental subproblem to efficiently operating the multiple UV systems is finding paths for UVs such that each target is visited by one of the UVs at least once while minimizing the final job completion time. When the UVs involved in an application are not identical to each other, differing in either structure or function or both, and located in distinctive initial locations, the problem is known as the min-max Multiple Depot Heterogeneous Traveling Salesman Problem (MDHTSP). When there exists only one UV—the simplest case—the problem becomes a traveling salesman problem (TSP). Thus, min-max MDHTSP is a generalized TSP and NP-hard [13].

The use of multiple UVs is preferred, especially in large environments. To find an optimal solution for these large sized problems, a large amount of computational effort is required. However, in many applications, the exact location of the vehicles and targets may not be known at the time of operation due to the uncertainty or limited capabilities of the sensors mounted on UVs. The research for path planning under sensor related issues are actively on-going [14,15,16,17]. For these cases, especially involving multiple heterogeneous UVs, having a good suboptimal solution within a short computation time could result in a higher efficiency rather than having an optimal solution after several days of computations. The approaches presented in this paper has been developed for such cases. In this study, as an initial step for solving min-max MDHTSP, the algorithms are developed to solve a TSP for systems involving two structurally heterogeneous UVs while focused on task allocation, finding an optimal assignment of targets for each vehicle and their optimal sequence of visit, and on routing, finding an optimal path for each vehicle in a given sequence. The problem is known as the two depot heterogeneous traveling salesman problem (TDHTSP). Based on previous work [18], the algorithm has been extended for solving the min-max objective by iteratively running the primal-dual algorithm for min-sum TDHTSP with some modification. In addition, we have also considered the case when travel costs are asymmetric with the direction of edges.

As research on the MDHTSP and multiple depot heterogeneous vehicle routing problems (MDHVRP) has advanced from a single agent problem, a majority of the research in literature solves the problem for the total travel costs of all agents to be minimized (min-sum). However, minimizing the maximum travel cost is essential in practical applications because it is directly related to the workload balance between agents and the reduction of job completion time. Few publications have dealt with the min-max MDHTSP or min-max MDHVRP. Franceschelli et al. proposed gossip algorithms for a class of heterogeneous multi-vehicle task assignment and routing problems [19]. Prasad and Choi presented an approximation algorithm for dealing with a task allocation problem for heterogeneous agents starting from a single depot [20].

There are also a few publications that solved min-max multiple depot vehicle routing problems of homogeneous vehicles. Stodola developed an algorithm for a min-max MDVRP based on the Ant Colony Optimization theory [21]. In Reference [22], Wang et al. proposed a heuristic that used a linear program (LP)-based approach as an initial solution and improved in further iterations. Some studies addressed the min-sum MDHTSP. Sundar and Rathinam developed an exact algorithm based on a branch-and-cut method while considering both structural and functional heterogeneity in Reference [23]. The transformation method of generalized MDHATSP into an ATSP was proposed in Reference [24]. A special type of problem called close open multi-depot mixed vehicle routing problem was dealt with in Reference [25], and a new mixed integer programming model and a hybrid metaheuristic were presented. In Table 1, a brief review of the related literature is presented. The categories “Heterogeneity(s)” and “Heterogeneity(f)” refer structural heterogeneity and functional heterogeneity, respectively.

As an initial step of solving min-max MDHTSP in time efficient manner, this paper made the following main contributions. First, the multiple depot heterogeneous traveling salesman problem that involves two heterogeneous vehicles is solved while considering cost symmetricity. Second, the primal-dual method [26] is applied for the min-max objective for the first time. The primal-dual method is an optimization technique that solves mixed integer program (MIP) problems with simple greedy algorithms. To apply primal-dual technique, the LP relaxation of MIP should be derived and its dual problem will be introduced. Then, by iteratively varying the dual variables, one can find an optimal or sub-optimal solution in time efficient manner. Lastly, relatively short computational time of the algorithm is shown through an implementation for different sizes of the problem.

The remainder of this paper is structured as follows: In Section 2, the problem is defined. In Section 3, a formulation of the TDHTSP where travel costs are symmetric is presented and a primal-dual based heuristic that solves the problem is proposed. In Section 4, the case where travel costs are asymmetric is considered. The proposed heuristics are implemented and the computational results are presented in Section 5. Section 6 consists some concluding remarks and future scope.

## 2. Problem Statement

As previously mentioned in Section 1, the Two Depot Heterogeneous Traveling Salesman Problem (TDHTSP) is considered in this article. Since only the structural heterogeneity is considered at this time, we assume that each vehicle has its own distinctive average running velocity and minimum turning radius. To handle this heterogeneity in an efficient manner, we assume that the faster vehicle has a smaller minimum turning radius compared to the slower vehicle. The faster vehicle is indexed as $r_{1}$ and the other one is indexed as $r_{2}$, such that $cost_{ij}^{1}\leq cost_{ij}^{2}\;\forall \left(i,j\right)$, where $cost_{ij}^{k}$ denotes the cost of traveling edge (i,j) using $r_{k}$. We also assume that the triangle inequality is satisfied for both UVs. In this article, two separate algorithms are developed for the cases when the travel costs are symmetric and asymmetric. Initially, the UVs are located at their distinctive depots. Each vehicle starts the route at its depot, visits a set of assigned targets, and finishes the route by turning back to the depot. The objective of this TDHTSP is to find a route for each vehicle such that each target is visited only once by one of the UVs while the maximum final tour cost is minimized.

When a set of *n* targets is given, the parameters and decision variables used in formulations, and the variables used in pseudo-codes can be described as follows:

Parameters:


$R=\{r_{1},r_{2}\}$
a set of UVs
$D=\{d_{1},d_{2}\}$
a set of depots
$T=\{t_{1},\cdots ,t_{n}\}$
a set of targets
$V_{k}=\left\{\left\{d_{k}\right\}\cup T\right\}$
a set of vertices for $r_{k}$
$E_{k}=\left\{\left(i,j\right),\;\forall i,j\in V_{k}\right\}$
a set of edges that connect all vertices in $V_{k}$
$C_{k}=\left\{cost_{ij}^{k},\forall \left(i,j\right)\in E_{k}\right\}$
a set of travel costs of all edges in $E_{k}$
$\delta_{k}\left(S\right)$
the subset of the edges of $E_{k}$ that have one end in *S* and the other end in $V_{k}\backslash S$
$\delta_{k}^{+}\left(S\right)$
the subset of the edges $\left(i,j\right)\in E_{k}$ such that $i\in V_{k}\backslash S$ and j∈S

Decision variables:


$x_{ij}^{k}$
the decision variable that represents whether edge (i,j) is used for the tour of $r_{k}$

$x_{ij}^{k}=\left\{\begin{array}{cc} 1 & \mathrm{if}\;\mathrm{edge}\;\left(i,j\right)\;\mathrm{is}\;\mathrm{traveled}\;\mathrm{by}\;r_{k}\\ 0 & \mathrm{otherwise} \end{array}\right.$

$z_{U}$
the decision variable that represents partition of targets in *T*

$z_{U}=\left\{\begin{array}{cc} 1 & \mathrm{if}\;\mathrm{set}\;U\;\mathrm{contains}\;\mathrm{all}\;\mathrm{the}\;\mathrm{vertices}\;\mathrm{not}\;\mathrm{visited}\;\mathrm{by}\;r_{1}\\ 0 & \mathrm{otherwise} \end{array}\right.$

*t*
the maximum travel cost

Variables used in algorithm pseudo-codes:


$TourCost_{k}$
the final tour cost of $r_{k}$ within a given partition of the targets
$Tour_{k}$
the final tour of $r_{k}$ within a given partition of the targets
$F_{k}$
a set of edges in the forest corresponding to $r_{k}$
$C_{k}$
a set of connected components in $F_{k}$
$Y_{k}\left(C\right)$
the dual variable of a set *C* in $C_{k}$, which keeps track of $\sum_{S\subseteq C}Y_{k}\left(S\right)$


$dual_{k}\left(v\right)$
the dual variable of a set *C* where v∈C, which keeps track of $\sum_{C:u\in C}Y_{k}\left(C\right)$
bound(C)
the dual variable of a set *C* in $C_{2}$, which keeps track of $\sum_{S\subseteq U}Y_{2}\left(S\right)$
$active_{k}\left(C\right)$
the activeness of set $C\in C_{k}$. $active_{k}\left(C\right)=1$ only if *C* is active.
Children(C)
the subsets of set $C\in C_{1}$ in $C_{2}$

As the definitions of the parameters, $\delta_{k}\left(S\right)$ contains all the edges crossing *S* and outside *S*, while $\delta_{k}^{+}\left(S\right)$ contains all the incoming edges to *S* from outside *S*. Moreover, only one subset *U* of *T* is allowed to have $z_{U}=1$, as $z_{U}$ is set to 1 only if *U* contains all of the vertices visited by $r_{2}$.

## 3. TDHTSP with Symmetric Travel Costs

### 3.1. Problem Formulation

When the travel costs are symmetric, i.e., $cost_{ij}^{k}=cost_{ji}^{k},\;\forall \left(i,j\right)\in E_{k},\;k=1,2$, we can formulate an mixed integer program as follows:

(1) $$\begin{array}{c} \;C_{IP_{1}}=\min t \end{array}$$

(2) $$\begin{array}{cc} \mathrm{subject}\;\mathrm{to}\\ \sum_{\left(i,j\right)\in \delta_{1}\left(S\right)}x_{ij}^{1}\geq \; & 2-2\sum_{T⊇U⊇S}z_{U}\;\forall S\subseteq T, \end{array}$$

(3) $$\begin{array}{cc} \sum_{\left(i,j\right)\in \delta_{2}\left(S\right)}x_{ij}^{2}\geq \; & 2\sum_{T⊇U⊇S}z_{U}\;\forall S\subseteq T, \end{array}$$

(4) $$\begin{array}{cc} \sum_{e\in \delta_{1}\left(i\right)}x_{ij}^{1}=\; & 2-2\sum_{T⊇U⊇\{i\}}z_{U}\;\forall i\in T, \end{array}$$

(5) $$\begin{array}{cc} \sum_{e\in \delta_{2}\left(i\right)}x_{ij}^{2}=\; & 2\sum_{T⊇U⊇\{i\}}z_{U}\;\forall i\in T, \end{array}$$

(6) $$\begin{array}{cc} t\geq & \sum_{\left\{i,j\right\}\in E_{k}}cost_{ij}^{k}\;x_{ij}^{k}\;k=1,2, \end{array}$$

(7) $$\begin{array}{cc} x_{ij}^{k}\in \; & \{0,1\}\;\forall i,j\in T,\;k=1,2, \end{array}$$

(8) $$\begin{array}{cc} x_{ij}^{k}\in \; & \left\{0,1,2\right\}\;\mathrm{for}\;i=d_{k},\;\forall j\in T,\;k=1,2, \end{array}$$

(9) $$\begin{array}{cc} \;z_{U}\in \; & \{0,1\}\;\forall U\subseteq T, \end{array}$$

(10) $$\begin{array}{cc} t\geq & 0. \end{array}$$

In this formulation, Equations (2) and (3) are the edge constraints that if there is at least one vertex in *S* that is not connected to the other depot, at least two edges must be chosen from $\delta_{k}\left(S\right)$ for the tour of $r_{k}$ for any S⊆T. Equations (4) and (5) are the degree constraints of each target. Equation (6) represents that *t* is the maximum travel cost, and Equations (7)–(9) are the integer constraints of decision variables. Equation (10) is the nonnegative constraint of *t*. Now, by relaxing the degree constraints and integer constraints, an LP can be formulated as shown below:

(11) $$\begin{array}{c} \;C_{LP_{1}}=\min t \end{array}$$

(12) $$\begin{array}{cc} \mathrm{subject}\;\mathrm{to}\\ \sum_{\left(i,j\right)\in \delta_{1}\left(S\right)}x_{ij}^{1}\geq \; & 2-2\sum_{T⊇U⊇S}z_{U}\;\forall S\subseteq T, \end{array}$$

(13) $$\begin{array}{cc} \sum_{\left(i,j\right)\in \delta_{2}\left(S\right)}x_{ij}^{2}\geq \; & 2\sum_{T⊇U⊇S}z_{U}\;\forall S\subseteq T, \end{array}$$

(14) $$\begin{array}{cc} t\geq & \sum_{\left\{i,j\right\}\in E_{k}}cost_{ij}^{k}\;x_{ij}^{k}\;k=1,2, \end{array}$$

(15) $$\begin{array}{cc} \;x_{ij}^{k}\geq & 0\;\forall \left(i,j\right)\in E_{k},\;k=1,2, \end{array}$$

(16) $$\begin{array}{cc} \;z_{U}\geq \; & 0\;\forall U\subseteq T, \end{array}$$

(17) $$\begin{array}{cc} t\geq \; & 0 \end{array}$$

To obtain the dual problem of the LP above, we introduce the dual variables, $Y_{1}\left(S\right)$, $Y_{2}\left(S\right)$, and $W_{k}$ for Equations (12)–(14), respectively. Then, the following dual problem can be introduced.

(18) $$\begin{array}{c} \;C_{dual_{1}}=\max2\sum_{S\subseteq T}Y_{1}\left(S\right) \end{array}$$

(19) $$\begin{array}{cc} \mathrm{subject}\;\mathrm{to}\\ \sum_{S:e\in \delta_{1}\left(S\right)}Y_{1}\left(S\right)\leq \; & cost_{ij}^{1}W_{1}\;\forall \left(i,j\right)\in E_{1}, \end{array}$$

(20) $$\begin{array}{cc} \sum_{S:e\in \delta_{2}\left(S\right)}Y_{2}\left(S\right)\leq \; & cost_{ij}^{2}W_{2}\;\forall \left(i,j\right)\in E_{2}, \end{array}$$

(21) $$\begin{array}{cc} \sum_{S\subseteq U}Y_{1}\left(S\right) & \leq \;\sum_{S\subseteq U}Y_{2}\left(S\right)\;\forall U\subseteq T, \end{array}$$

(22) $$\begin{array}{cc} W_{1}+W_{2}\leq \; & 1 \end{array}$$

(23) $$\begin{array}{cc} Y_{k}\left(S\right)\geq \; & 0\;\forall S\subseteq T,\;k=1,2, \end{array}$$

(24) $$\begin{array}{cc} W_{k}\geq \; & 0\;k=1,2. \end{array}$$

In the proposed heuristic, the dual variables $Y_{1}\left(S\right),Y_{2}\left(S\right)$ can be interpreted as the costs that each set of *S* is willing to pay to be connected to the corresponding depots. The dual variable $W_{k}$ can be interpreted as the weight that prioritizes one of the UVs, i.e., if $W_{1}>W_{2}$, the algorithm will give priority to $r_{2}$, and vice versa. By adjusting the values of $W_{k}$ while maintaining the cost condition of $W_{1}cost_{ij}^{1}\leq W_{2}cost_{ij}^{2}\;\forall \left(i,j\right)$ to be satisfied all times, we may be able to decrease the maximum tour cost in the proposed algorithm. With a fixed $W_{k}$, we can obtain a heterogeneous spanning forest (HSF) that connects each vertex to one of the depots by using this dual problem. The obtained HSF is utilized as a target assignment, and the routes can be derived within the partitions using existing algorithms.

### 3.2. A Heuristic for the TDHTSP

The pseudo-codes of the heuristic for TDHTSP is presented in Algorithms 1 and 2. In Algorithm 1, to reduce the maximum travel cost, we iteratively run Algorithm 2 by varying the values of $W_{1}$ and $W_{2}$. As presented in Algorithm 1, we at first set $W_{1}$ and $W_{2}$ to be equal to 0.5. Based on the initial routing result, we increase or decrease $W_{1}$ with a small value ϵ≤0.1 and set $W_{2}$ as $1-W_{1}$ so that the constraint in Equation (22) is tight all the time. With updated $W_{k}$ values, the routes are newly derived and we determine whether any reduction exists in *t*. Iteration stops when either the other vehicle has the maximum tour cost or there exists any violation in the cost condition of $W_{1}cost_{ij}^{1}\leq W_{2}cost_{ij}^{2}\;\forall \left(i,j\right)$.

With fixed values for $W_{1}$ and $W_{2}$, we derive a task allocation for the UVs using the primal-dual heuristic that solves an HSF based on the dual problem in Equations (18)–(24) that is presented in Algorithm 2. Initially, each set contains one vertex and each tree $F_{k}$ is empty. The sets containing targets are active, and the sets containing depots are inactive. All dual variables are set to zero, and all vertices are unmarked. In the main loop, the algorithm consistently increases the dual variables of the active sets by an amount that makes one of dual constraints in Equations (19)–(21) tight. If one of the constraints in Equations (19) or (20) becomes tight, the corresponding edge is added to the tree $F_{k}$. The two sets connected through the newly added edge are now combined into a new set. If the new set does not contain the depot, it becomes an active set. Otherwise, the new set is deactivated. If k=1 and the new set contains $d_{1}$, all children (the subsets in $C_{2}$) of the newly formed set are also deactivated. If one of the constraints in Equations (21) becomes tight, the corresponding set is deactivated and the vertices in the set are marked. The iteration terminates when all sets in $C_{1}$ become inactive.

**Figure 1:**
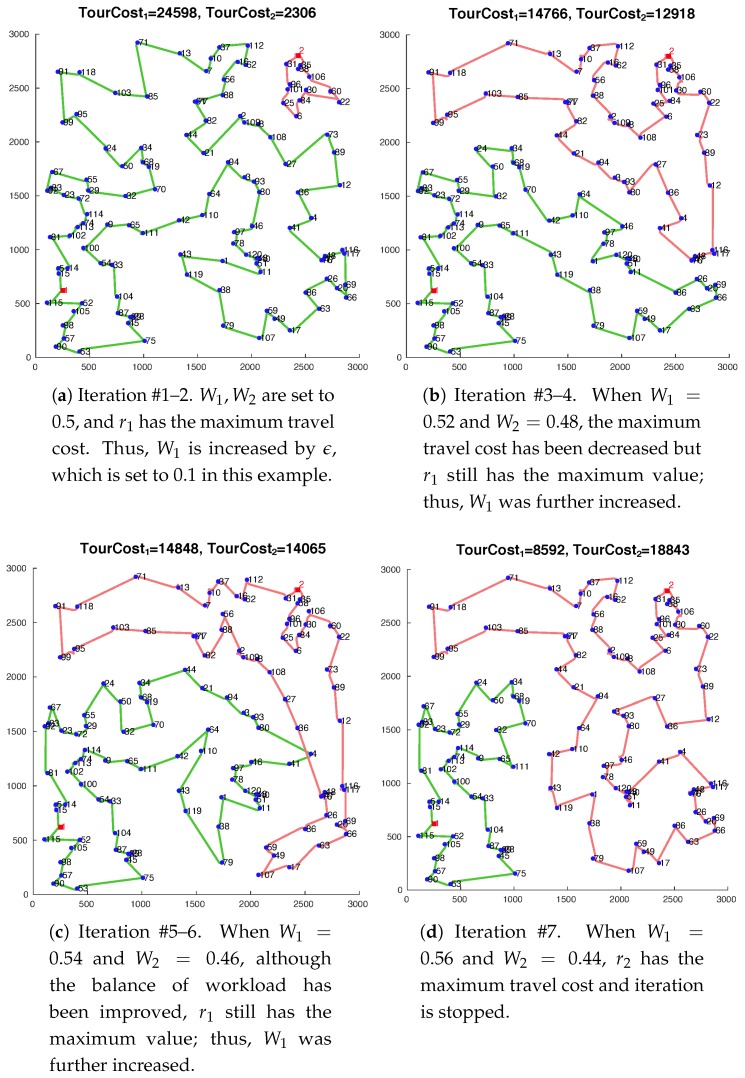
Routes produced with Algorithm 1 for an instance of 120 targets in a 3000×3000 space with symmetric costs: As the routes in (**b**) have the lowest maximum travel cost, 14,766, it has been chosen for the final routes.

At this point, most primal-dual algorithms that solve min-sum TSPs perform reverse-deleting as pruning steps after the main loop is terminated because the edge added at the *i*-th iteration has a smaller cost than the edge added at the (i+1)-th iteration for any *i* in the loop. Deletion of the unnecessary edges in a reverse order would reduce the total sum of the travel costs. However, as we are dealing with a min-max problem, we need to consider the balance of workload. Thus, instead of reverse-deleting, we perform pruning steps we designed in the proposed heuristic. Once the main loop is terminated, we sort the targets into three partitions: $P_{1},P_{2}$, and $P_{c}$. Each $P_{k}$ for k=1,2 contains the vertices that are only connected to $d_{k}$, while $P_{c}$ contains the vertices that are connected to both $d_{1}$ and $d_{2}$. For $P_{1}$ and $P_{2}$, we find the minimum spanning trees $T_{1}$ and $T_{2}$, respectively. If $P_{k}$ only contains its depot, $T_{k}$ is set to be empty. Then, we distribute the targets in $P_{c}$ to $P_{1}$ and $P_{2}$ depending on the current workload. Until all targets in $P_{c}$ are distributed, the algorithm finds edges $e_{k}$ for k=1,2 that connect any vertex in $P_{k}$ to one of the targets in $P_{c}$ with the minimum cost. Depending on the current total edge costs in $T_{k}$, the edge is added to the tree with a lower cost. The corresponding vertex is removed from $P_{c}$ and added to $P_{k}$. When $P_{c}$ becomes empty, each of the targets will be connected to only one of the depots in either $T_{1}$ or $T_{2}$. Once partitioning is finished, a tour for each partition can be found using existing algorithms, i.e., LKH [27] in our implementation. In Figure 1, the routes of each iteration of Algorithm 1 for an instance with 120 targets has been presented. Finally, proof of the feasibility of the proposed heuristic for TDHTSP and running time analysis are presented in Lemma 1.

**Algorithm 1** A heuristic for min-max TDHTSP1:$W_{1}=W_{2}=0.5$;2:Determine a heterogeneous spanning forest using the proposed primal-dual heuristic.3:
**for**
k=1,2
**do**
4: Let the connected targets reachable from the depot be a partition and label it as $P_{k}$.5:
**end for**
6:For k=1,2, derive $TourCost_{k}$ and $Tour_{k}$ for $r_{k}$ within $P_{k}$ (using an existing routing algorithm).7:
$G\leftarrow \max(TourCost_{k})$
8:
**if**
$TourCost_{1}\geq TourCost_{2}$
**then**
9: **while**
$TourCost_{1}\geq TourCost_{2}$
**do**10:  $W_{1}\leftarrow W_{1}+\epsilon ;\;W_{2}\leftarrow 1-W_{1}$11:  Redetermine a target assignment using the proposed primal-dual heuristic and obtain the partitions $P_{k}^{\prime }$.12:  **for**
k=1,2
**do**13:   Derive $TourCost_{k}^{\prime }$ and $Tour_{k}^{\prime }$ within $P_{k}^{\prime }$.14:  **end for**15:  **if**
$Tour^{\prime }$ is infeasible **then**16:   **break,** {*comment:*
$∵W_{1}\times cost_{ij}^{1}>W_{2}\times cost_{ij}^{2}$ for some (i,j)}17:  **else**18:   $G^{\prime }\leftarrow \max\left(TourCost_{k}^{\prime }\right)$19:   **if**
$G^{\prime }<G$
**then**20:    $G\leftarrow G^{\prime }$21:    **for**
k=1,2
**do**22:     $TourCost_{k}\leftarrow TourCost_{k}^{\prime };\;Tour_{k}\leftarrow Tour_{k}^{\prime }$23:    **end for**24:   **end if**25:  **end if**26: **end while**27:
**else**
28: **while**
$TourCost_{1}\leq TourCost_{2}$
**do**29:  $W_{1}\leftarrow W_{1}-\epsilon ;\;W_{2}\leftarrow 1-W_{1}$30:  Redetermine a target assignment using the proposed primal-dual heuristic and obtain the partitions $P_{k}^{\prime }$.31:  **for**
k=1,2
**do**32:   Derive $TourCost_{k}^{\prime }$ and $Tour_{k}^{\prime }$ within $P_{k}^{\prime }$.33:  **end for**34:  $G^{\prime }\leftarrow \max\left(TourCost_{k}^{\prime }\right)$35:  **if**
$G^{\prime }<G$
**then**36:   $G\leftarrow G^{\prime }$37:   **for**
k=1,2
**do**38:    $TourCost_{k}\leftarrow TourCost_{k}^{\prime };\;Tour_{k}\leftarrow Tour_{k}^{\prime }$39:   **end for**40:  **end if**41: **end while**42:
**end if**
43:
**return**
$Tour_{k},TourCost_{k}$


**Algorithm 2** Primal-dual heuristic for finding an HSF1:Initialization2:$F_{k}\leftarrow \emptyset$, $C_{k}\leftarrow \left\{\left\{v\right\}:v\in V_{k}\right\}$ for k=1,23:All vertices are unmarked.4:All dual variables are set to zero.5:$active_{k}\left(\left\{v\right\}\right)\leftarrow 1$, $\forall v\in V_{k}$, for k=1,26:$active_{k}\left(\left\{d_{k}\right\}\right)\leftarrow 0$, for k=1,27:Main loop8:**while** there exists any active component in $C_{1}$
**do**9: **for**
k=1,2
**do**10:  Find an edge $e_{k}=\left(i,j\right)\in E_{k}$ with $i\in C_{i},j\in C_{j}$ where $C_{i},C_{j}\in C_{k},C_{i}\neq C_{j}$ that minimizes $\varepsilon_{k}=\frac{\left\{W_{k}\times cost_{ij}^{k}-dual_{k}\left(i\right)-dual_{k}\left(j\right)\right\}}{active_{k}\left(C_{i}\right)+active_{k}\left(C_{j}\right)}$.11: **end for**12: Let $ℜ:=\{R:active_{k}\left(R\right)=1,\mathrm{One}\;\mathrm{of}\;\mathrm{subsets}\;\mathrm{of}\;R\;\mathrm{contains}\;d_{2},\;\forall R\in C_{1}\}$.13: Find $\bar{R}\in ℜ$ that minimizes $\varepsilon_{3}=bound\left(\bar{R}\right)-Y_{1}\left(\bar{R}\right)$14: $\varepsilon_{min}=\min\left(\varepsilon_{1},\varepsilon_{2},\varepsilon_{3}\right)$15: **for**
k=1,2
**do**16:  **for**
$C\in C_{k}$
**do**17:   $Y_{k}\left(C\right)\leftarrow Y_{k}\left(C\right)+\varepsilon_{min}\times active_{k}\left(C\right)$18:   $dual_{k}\left(v\right)\leftarrow dual_{k}\left(v\right)+\varepsilon_{min}\times active_{k}\left(C\right),\forall v\in C$19:   **if**
k=1
**then**20:    $bound\left(C\right)\leftarrow bound\left(C\right)+\varepsilon_{min}\left|Children\left(C\right)\right|$21:   **end if**22:  **end for**23: **end for**24: **if**
$\varepsilon_{min}=\varepsilon_{k}$ for k=1 or 2 **then**25:  $F_{k}\leftarrow \left\{e_{k}\right\}\cup F_{k}$26:  $C_{k}\leftarrow C_{k}\cup \left\{C_{i}\cup C_{j}\right\}-C_{i}-C_{j}$27:  $Y_{k}\left(\left\{C_{i}\cup C_{j}\right\}\right)=Y_{k}\left(C_{i}\right)+Y_{k}\left(C_{j}\right)$28:  **if**
k=1
**then**29:   $Bound\left(C_{i}\cup C_{j}\right)\leftarrow bound\left(C_{i}\right)+bound\left(C_{j}\right)$30:  **end if**31:  **if**
$d_{k}\in \left\{C_{i}\cup C_{j}\right\}$
**then**32:   $active_{k}\left(C_{i}\cup C_{j}\right)\leftarrow 0$33:   **if**
k=1
**then**34:    $active_{2}\left(C\right)\leftarrow 0\;\forall C\in Children\left(C_{i}\cup C_{j}\right)$35:   **end if**36:  **else**37:   $active_{k}\left(C_{i}\cup C_{j}\right)\leftarrow 1$38:  **end if**39: **else**40:  $active_{1}\left(\bar{C}\right)\leftarrow 0$41:  Mark all of the vertices of $\bar{C}$ with the label $\bar{C}$.42: **end if**43:
**end while**
44:Pruning45:
$P_{k}\leftarrow \mathrm{all}\;\mathrm{vertices}\;\mathrm{that}\;\mathrm{are}\;\mathrm{only}\;\mathrm{connected}\;\mathrm{to}\;d_{k}\;\mathrm{for}\;k=1,2$
46:
$P_{c}\leftarrow \mathrm{the}\;\mathrm{vertices}\;\mathrm{that}\;\mathrm{are}\;\mathrm{connected}\;\mathrm{to}\;\mathrm{both}\;d_{1}\;\mathrm{and}\;d_{2}$
47:Let $T_{k}$ be the minimum spanning tree of $P_{k}\;\mathrm{for}\;k=1,2$.48:Let $C(T_{k})$ be the sum of edge costs present in $T_{k}$49:**while**$P_{c}$ is not empty **do**50: Find the shortest edge $e_{k}$ that connects a vertex in $P_{c}$ and a vertex in $P_{k}$ for each *k*.51: **if**
$C\left(T_{1}\right)\leq C\left(T_{2}\right)$
**then**52:  Add $e_{1}$ to $T_{1}$, remove the corresponding vertex from $P_{c}$, and add it to $P_{1}$.53: **else**54:  Add $e_{2}$ to $T_{2}$, remove the corresponding vertex from $P_{c}$ and add it to $P_{2}$.55: **end if**56:
**end while**

**Lemma** **1.** *The proposed heuristic (in Algorithms 1 and 2) produces a feasible solution for min-max TDHTSP. Each of the vertices is reachable from one of the depots, and every vertex appears only once in the routes. In addition, Algorithm 2 runs in polynomial time.*

**Proof.**  During the main loop in Algorithm 2, the loop terminates when all the components of $C_{1}$ become inactive. There exists only two cases that can happen for deactivation. First, a set *C* is connected to its depot, and second, one of the constraints in Equation (21) becomes tight. However, the second case can happen only if the subsets of *C* in $C_{2}$ is connected to $d_{2}$. Thus, the only possible way for termination is if each of the targets in *T* is connected to either $d_{1}$ or $d_{2}$. Because we make sure that all vertices are connected to one of the components in pruning steps, the proposed heuristic produces a feasible solution for the given problem.

In addition, because there are at most *n* components for each robot at any point in time of running Algorithm 2, each iteration will have O(n) searches, yielding a time bound of O(nlog(n)) per iteration or $O(n^{2}log\left(n\right))$ for the entire loop. Thus, Algorithm 2 runs in polynomial time.  □

## 4. TDHTSP with Asymmetric Travel Costs

### 4.1. Problem Formulation

When the travel costs are asymmetric, i.e., $cost_{ij}^{k}\neq cost_{ji}^{k}$, the directions of the edges need to be considered to generate tours. This problem is known as the two depot heterogeneous asymmetric traveling salesman problem (TDHATSP). We can formulate the following LP by considering only the incoming edges.

(25) $$\begin{array}{c} \;C_{LP_{2}}=\min t \end{array}$$

(26) $$\begin{array}{cc} \mathrm{subject}\;\mathrm{to}\\ \sum_{\left\{i,j\right\}\in \delta_{1}^{+}\left(S\right)}x_{ij}^{1}\geq \; & 1-\sum_{T⊇U⊇S}z_{U}\;\forall S\subseteq T, \end{array}$$

(27) $$\begin{array}{cc} \sum_{\left\{i,j\right\}\in \delta_{2}^{+}\left(S\right)}x_{ij}^{2}\geq & \sum_{T⊇U⊇S}z_{U}\;\forall S\subseteq T, \end{array}$$

(28) $$\begin{array}{cc} t\geq & \sum_{\left\{i,j\right\}\in E_{k}}cost_{ij}^{k}\;x_{ij}^{k}\;k=1,2, \end{array}$$

(29) $$\begin{array}{cc} x_{ij}^{k}\geq \; & 0\;\forall \left(i,j\right)\in E_{k},\;k=1,2, \end{array}$$

(30) $$\begin{array}{cc} z_{U}\geq \; & 0\;\forall U\subseteq T, \end{array}$$

(31) $$\begin{array}{cc} t\geq \; & 0 \end{array}$$

Similar to the symmetric case in Section 3.1, by introducing the dual variables $Y_{1}^{+}\left(S\right)$, $Y_{2}^{+}\left(S\right)$ and $W_{k}^{+}$ for Equations (26)–(28), the dual problem of the TDHATSP can be formulated as shown below:

(32) $$\begin{array}{c} C_{dual_{2}}=\max\sum_{S\subseteq T}Y_{1}^{+}\left(S\right) \end{array}$$

(33) $$\begin{array}{cc} \mathrm{subject}\;\mathrm{to}\\ \sum_{S:e\in \delta_{1}^{+}\left(S\right)}Y_{1}^{+}\left(S\right)\leq \; & cost_{ij}^{1}W_{1}^{+}\;\forall \left(i,j\right)\in E_{1}, \end{array}$$

(34) $$\begin{array}{cc} \sum_{S:e\in \delta_{2}^{+}\left(S\right)}Y_{2}^{+}\left(S\right)\leq \; & cost_{ij}^{2}W_{2}^{+}\;\forall \left(i,j\right)\in E_{2}, \end{array}$$

(35) $$\begin{array}{cc} \sum_{S\subseteq U}Y_{1}^{+}\left(S\right)\leq \; & \sum_{S\subseteq U}Y_{2}^{+}\left(S\right)\;\forall U\subseteq T, \end{array}$$

(36) $$\begin{array}{cc} W_{1}^{+}+W_{2}^{+}\leq \; & 1 \end{array}$$

(37) $$\begin{array}{cc} Y_{k}^{+}\left(S\right)\geq \; & 0\;\forall S\subseteq T,\;k=1,2, \end{array}$$

(38) $$\begin{array}{cc} W_{k}^{+}\geq \; & 0\;k=1,2. \end{array}$$

### 4.2. A Heuristic for the TDHATSP

The difference between the symmetric and asymmetric cases lies in the primal-dual heuristic for obtaining partitions. For asymmetric case, we apply the primal-dual technique to derive a heterogeneous directed spanning forest (HDSF). During iteration to find the best $W_{k}$ values, we use the same algorithm presented in Algorithm 1 as we did in Section 3. The algorithm for obtaining an HDSF is presented in Algorithm 3.

**Algorithm 3** Primal-dual heuristic for finding an HDSF1:Initialization2:$F_{k}\leftarrow \emptyset$, $C_{k}\leftarrow \left\{\left\{v\right\}:v\in V_{k}\right\}$ for k=1,23:All the vertices are unmarked.4:All the dual variables are set to zero.5:$active_{k}\left(\left\{v\right\}\right)\leftarrow 1$, $\forall v\in V_{k}$, for k=1,26:$active_{k}\left(\left\{d_{k}\right\}\right)\leftarrow 0$, for k=1,27:Main loop8:**while** there exists any active component in $C_{1},C_{2}$
**do**9: **for**
*k* = 1, 2 **do**10:  Find an edge $e_{k}=\left(i,j\right)\in E_{k}$ with $i\in C_{i},j\in C_{j}$, where $C_{i},C_{j}\in C_{k},C_{i}\neq C_{j}$ that minimizes $\varepsilon_{k}=\frac{W_{k}^{+}cost_{ij}^{k}-dual_{k}\left(j\right)}{active_{k}\left(C_{j}\right)}$11: **end for**12: Let the corresponding $C_{j}\in C_{k}$ be $S_{k}$, while $S=\{S_{1},S_{2}\}$ satisfies $S_{1}⊇S_{2}$ and $S_{1},S_{2}$ are active.13: $F_{k}\leftarrow \left\{e_{k}\right\}\cup F_{k}$14: Increase the dual variables of $S_{k}$ by $\varepsilon_{k}$.15: **if**$e_{k}$ forms a new strongly connected component and the component is not reachable from $d_{k}$
**then**16:  Let the strongly connected component be an active component.17: **else if**
$e_{k}$ makes any vertex $v\in S_{k}$ reachable from $d_{k}$
**then**18:  Let the depot and all vertices that are reachable from the depot be an inactive component.19:  **if**
$e_{k}=e_{1}$
**then**20:   Deactivate all subsets of this component in $C_{2}$.21:  **else**22:   Mark all vertices in the supersets of this component in $C_{1}$. Deactivate it if the corresponding component consists of all marked vertices.23:  **end if**24: **else**25:  Deactivate $S_{k}$.26: **end if**27: **if** there exists no $S=\{S_{1},S_{2}\}$ that can be chosen that satisfies the given conditions and there exists any inactive set without an incoming edge that is not connected to the depot **then**28:  Pick an inactive component for each *k* that consists of marked vertices that have incoming or outgoing edges. Combine those connected components until the new component does not have any incoming edges.29: **end if**30:
**end while**
31:Pruning32:
$P_{k}\leftarrow \mathrm{all}\;\mathrm{vertices}\;\mathrm{that}\;\mathrm{are}\;\mathrm{only}\;\mathrm{reachable}\;\mathrm{from}\;d_{k}\;\mathrm{for}\;k=1,2$
33:
$P_{c}\leftarrow \mathrm{the}\;\mathrm{vertices}\;\mathrm{that}\;\mathrm{are}\;\mathrm{reachable}\;\mathrm{from}\;\mathrm{both}\;d_{1}\;\mathrm{and}\;d_{2}$
34:Let $T_{k}$ be the minimum directed spanning tree of $P_{k}\;\mathrm{for}\;k=1,2$.35:Let $C(T_{k})$ be the sum of the edge costs present in $T_{k}$.36:**while**$P_{c}$ is not empty **do**37: Find the shortest edge $e_{k}$ that makes a vertex in $P_{c}$ reachable from the vertices in $P_{k}$ for each *k*.38: **if**
$C\left(T_{1}\right)\leq C\left(T_{2}\right)$
**then**39:  Add $e_{1}$ to $T_{1}$, remove the corresponding vertex from $P_{c}$, and add it to $P_{1}$.40: **else**41:  Add $e_{2}$ to $T_{2}$, remove the corresponding vertex from $P_{c}$, and add it to $P_{2}$.42: **end if**43:
**end while**


Similar to the symmetric case, once $W_{1}$ and $W_{2}$ are fixed, we derive a target assignment using a primal-dual heuristic that solves an HDSF based on the dual problem in Equations (32)–(38). Initially, each set contains one vertex and each tree $F_{k}$ is empty. The sets containing targets are active, while the sets containing depots are inactive. All vertices are unmarked, and all dual variables are set to zero. In the main loop, the algorithm iteratively searches for the dual variables that make one of the dual constraints in Equations (33) and (34) tight with the minimum increase, where the corresponding set in $C_{1}$ and at least one of its subsets is active. The corresponding new edge is added to its tree $F_{k}$. There are three cases that can occur. First, if the new edge forms any new strongly connected component that is reachable from its depot, let the strongly connected component be a new active component. Second, if the new edge forms a new strongly connected component that is reachable from $d_{k}$, let the depot and all reachable vertices be a new inactive component. If $e_{1}$ has the minimum value, all subsets in $C_{2}$ are deactivated. If $e_{2}$ is the minimum value, all vertices in the supersets of the component in $C_{1}$ are marked and deactivated. This step ensures that the third dual constraint in Equation (35) holds all of the time during iteration. Third, if neither the first nor second case occurs, deactivate the corresponding set. Once the sets are updated, the algorithm checks whether there exists any active set that has at least one active subset that can be chosen. If not, find an inactive set that has no incoming edges and is not reachable from the depot. According to the design of the algorithm, those sets should have at least one connected set that contains marked vertices. Thus, we form a new active component by combining those sets until the new set does not have any incoming edges. Once the main loop is terminated, we perform the pruning steps as we did for the symmetric case in Algorithm 2. Since the edges have directions, we form the minimum directed spanning trees for each partition $P_{k}$ and iteratively distribute the vertices in $P_{c}$ to the one that has the lower tree cost. Now, the feasibility of the proposed heuristic for TDHATSP is shown in Lemma 2. Because the running time analysis is very similar with symmetric case, the proof is omitted.

**Lemma** **2.** *The proposed heuristic (in Algorithm 1 and 3) produces a feasible solution for min-max TDHTSP. Each of the vertices is reachable from one of the depots, and every vertex appears only once in the routes.*

**Proof.**  During the main loop in Algorithm 3, the loop terminates when all components become inactive. There are three cases that can happen for deactivation: (1) The edge added to the forest does not form any new strongly connected component, and no vertices in the component are reachable from one of the depots; (2) the component has become reachable from its depot; and (3) one of its subsets or supersets becomes reachable from its depot. The vertices would become reachable from one of the depots if (2) or (3) happens at least once at the time of termination.

Assume that only one active component has left for all $C_{k}$ after having case (1) for all iterations. Then, there exist only two cases that can happen in the next iteration. Either the component becomes reachable from one of the depots and terminates the loop or a new active strongly connected component is formed and continues. This would be true until there exists only one large active component that containing all the vertices left except the depot, which implies the latter case cannot happen at the next iteration. Thus, at least one edge starting from one of the depots should be added before the termination. This implies that, at the time of termination, each vertex is connected to at least one of the depots. During the pruning steps, we make sure that all vertices are connected to only one of the depots while distributing vertices in $P_{c}$. Thus, even if there existed the vertices that were connected to both depots, only one depot would be left at the end of the algorithm. Hence, the algorithm produces a feasible solution for TDHTSP.  □

## 5. Implementation

**Figure 2:**
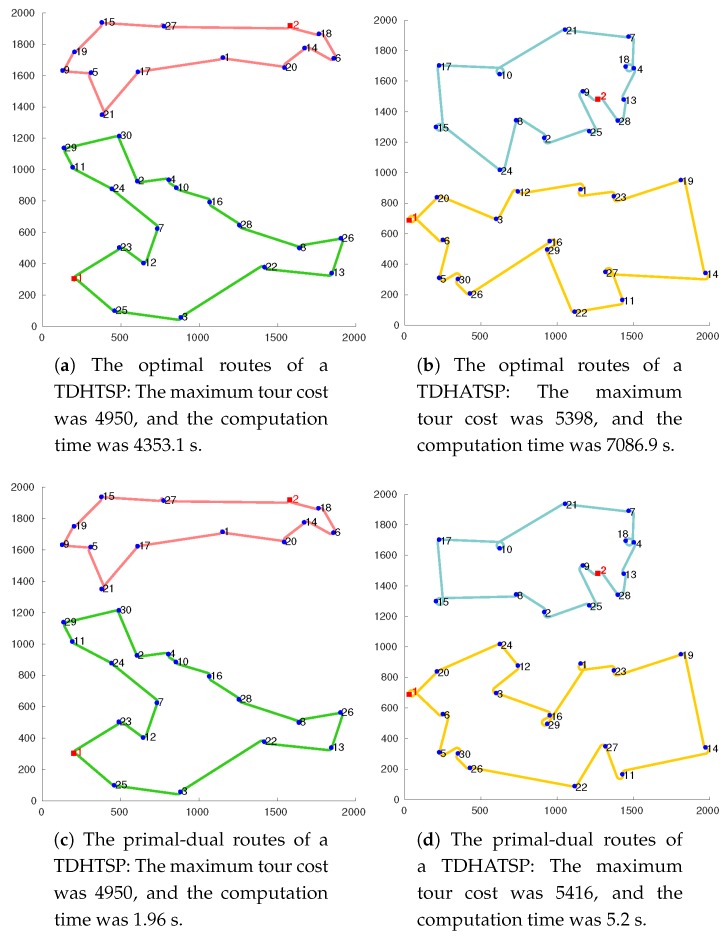

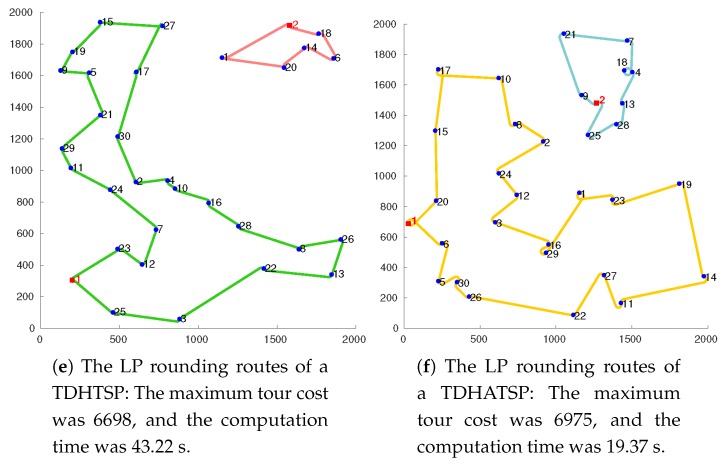
Routes generated by three different algorithms for instances of the cost-symmetric problem (**left**) and cost-asymmetric problem (**right**) with 30 targets in a 2000×2000 space.

In this section, we show the computational aspects of the proposed algorithms. All computational experiments were performed with a PC equipped with an Intel^®^Core™ i5-7600 CPU running at 3.5 GHz with 8 GB RAM. Simulations were performed on a virtual square space with a size of 2000×2000 units. The proposed algorithms were implemented using MATLAB software and run repeatedly for instances with varying problem sizes. The number of targets was set to 20, 30, 40, 60, and 80. We evaluated 50 instances for each problem size. The locations of the targets and depots were randomly generated with a uniform distribution. We assumed that both $r_{1}$ and $r_{2}$ have the motion of a Reeds–Shepp vehicle [28] for the cost-symmetric cases and the motion of a Dubins vehicle [29] for the cost-asymmetric cases. To ensure that the cost inequality is satisfied, we set the average velocity of movement $v_{k}$ to be $v_{1}>v_{2}$ and the minimum turning radius $\rho_{k}$ to be $\rho_{1}<\rho_{2}$. Due to the motion constraints of the robots, we generated the locations of targets to stay within the margins of $\rho_{1}$. The costs to travel between the locations were set to the minimum travel time between the locations by dividing the Reeds–Shepp curve [30] and Dubins curve [29] by the average velocity of each vehicle. Specifically, we applied $\rho_{1}=20,\;\rho_{2}=25$, $v_{1}=1.2,$ and $v_{2}=0.9$ in the simulations. Example routes for 30 targets produced by three different algorithms for both symmetric and asymmetric cases are presented in Figure 2. In the examples, the heuristics produced the optimal and near optimal solutions within much shorter computation times.

**Figure 3:**
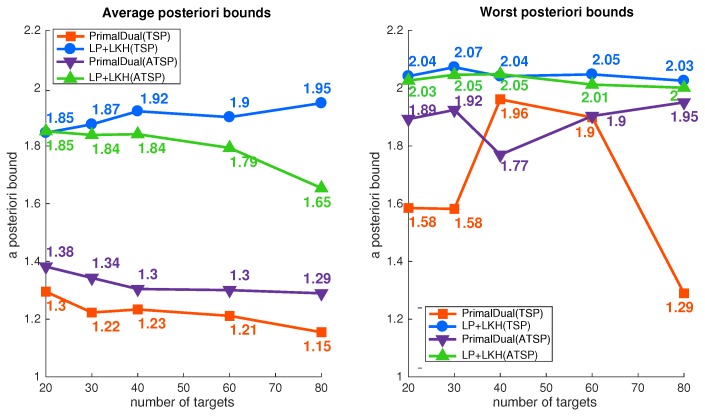
Posteriori bounds of 50 instances for each problem size.

To ascertain the performance, LP relaxation solutions were used to find a posteriori bounds. To solve the LP relaxation, we used a multi-commodity flow formulation on CPLEX [31]. We compared the solutions for the same instances with an LP rounding method, which solves the LP relaxation and assigns each target to the one has the maximum value of the partitioning decision variable. To solve the routing problem within the partition, LKH [32] was used for both Algorithm 2 and Algorithm 3. The results of the computational evaluation is presented in Figure 3 and Table 2 and Table 3.

### Discussion of Implementation Results

The average and worst a posteriori bounds of each problem size for both cost-symmetric and cost-asymmetric cases are presented in Figure 3. A posteriori bound was calculated by $Cost_{A}\left(I\right)÷Cost_{LP}\left(I\right)$, where $Cost_{A}\left(I\right)$ represents the cost of the solution obtained through algorithm A of instance *I* and $Cost_{LP}\left(I\right)$ represents LP relaxation cost for instance *I*. Despite the fact that LP rounding usually produced better quality solutions for the min-sum MDHTSP and min-sum MDHATSP [33], the proposed heuristic produced better posteriori bounds for all problem sizes with the min-max objective. For both the symmetric and asymmetric problems, the proposed methods had average posteriori bounds less than 1.4, while LP rounding had bounds greater than 1.6. The worst a posteriori bounds of LP rounding were larger than two for all sizes, while those for the proposed heuristics were less than two.

The average and worst computation times of cost-symmetric and cost-asymmetric cases are summarized in Table 2 and Table 3, respectively. In both tables, there exists a huge gap in the computation time between the two algorithms, especially for large-sized problems. For example, the LP rounding method required average of 39,891.46 s, i.e., longer than 11 h, while the proposed heuristic takes approximately 20 s to solve the cost-symmetric problems of 80 targets. As presented in blue and red in Table 2 and Table 3, the average computation time was decreased to 0.0005% and 0.001% compare to the LP rounding method for the symmetric and asymmetric cases of 80 targets, respectively, while maintaining better average solution qualities. Therefore, the proposed heuristics are better suitable for applications that require good approximate solutions within a short period of time to increase profits.

## 6. Conclusions

In this research, we proposed heuristics based on a primal-dual technique for the job allocation and routing of a two-UV system with structural heterogeneity while minimizing the last job completion time. The formulations of MIP, LP, and dual problems were presented. Using the dual problems, the heuristics were designed to iteratively run primal-dual algorithms by varying the $W_{k}$ values, which implies the priority of *k*th vehicle. Through implementation, the effectiveness of our proposed algorithms has been shown.

Our heuristics have limitations for guarantees of worst solution quality, limited number of vehicles, and limited heterogeneity. However, we believe that our heuristics are a good original idea that can solve the min-max MDHTSP in a time-efficient manner while considering additional constraints that increase the complexity of the problem. As future work, we aim to solve the mim-max MDHTSP and min-max MDHATSP while considering both structural and functional heterogeneity with lighter computational loads.

## Figures and Tables

**Table 1:** An overview of the literature of related problems.

	Depots	Heterogeneity(s)	Heterogeneity(f)	Objective	Methodology	Cost
This Paper	Two	Yes	No	Min-Max	Primal-Dual	Symmetric and Asymmetric
[18]	two	Yes	No	min-sum	primal-dual	symmetric
[19]	multiple	Yes	No	min-max	gossip algorithm	symmetric
[20]	single	No	Yes	min-max	5-approx algo.	symmetric
[21]	multiple	No	No	min-max	ant colony opt.	symmetric
[22]	multiple	No	No	min-max	LP based heuristic	symmetric
[23]	multiple	Yes	Yes	min-sum	branch and cut	asymmetric
[24]	multiple	Yes	No	min-sum	transformation	asymmetric
[25]	multiple	No	Yes	min-sum	hybrid GA	symmetric

**Table 2:** The computation time in seconds for the TDHTSP.

	Average		Worst	
No. of Jobs	LP Rounding	Primal-Dual	LP Rounding	Primal-Dual
20	2.11	0.81	2.91	1.61
30	31.13	1.79	41.23	2.35
40	429.61	3.30	867.66	5.26
60	5623.48	8.62	8470.95	11.72
80	39,891.46	19.65	63,239.30	31.02

**Table 3:** The computation time in seconds for the TDHATSP.

	Average		Worst	
No. of Jobs	LP Rounding	Primal-Dual	LP Rounding	Primal-Dual
20	1.59	1.23	2.38	3.28
30	17.70	4.80	22.11	12.31
40	237.06	4.86	345.81	12.79
60	3634.76	16.00	10006.30	46.08
80	30,329.19	36.21	41,330.20	99.87

## References

[1] Reinecke M., Prinsloo T. The influence of drone monitoring on crop health and harvest size. Proceedings of the 2017 1st International Conference on Next Generation Computing Applications (NextComp).

[2] Malveaux C., Hall S.G., Price R. (2014). Using Drones in Agriculture: Unmanned Aerial Systems for Agricultural Remote Sensing Applications. Am. Soc. Agric. Biol. Eng..

[3] Kanistras K., Martins G., Rutherford M.J., Valavanis K.P. A survey of unmanned aerial vehicles (UAVs) for traffic monitoring. Proceedings of the 2013 International Conference on Unmanned Aircraft Systems (ICUAS).

[4] Yuan C., Zhang Y., Liu Z. (2015). A survey on technologies for automatic forest fire monitoring, detection, and fighting using unmanned aerial vehicles and remote sensing techniques. Can. J. For. Res..

[5] Casbeer D.W., Kingston D.B., Beard R.W., McLain T.W. (2006). Cooperative forest fire surveillance using a team of small unmanned air vehicles. Int. J. Syst. Sci..

[6] Mendonça R., Marques M.M., Marques F., Lourenço A., Pinto E., Santana P., Coito F., Lobo V., Barata J. A cooperative multi-robot team for the surveillance of shipwreck survivors at sea. Proceedings of the OCEANS 2016 MTS/IEEE Monterey.

[7] Jia Z., Yu J., Ai X., Xu X., Yang D. (2018). Cooperative multiple task assignment problem with stochastic velocities and time windows for heterogeneous unmanned aerial vehicles using a genetic algorithm. Aerosp. Sci. Technol..

[8] Calhoun G., Ruff H., Behymer K., Frost E. (2018). Human-autonomy teaming interface design considerations for multi-unmanned vehicle control. Theor. Issues Ergon. Sci..

[9] Murphy R.R., Kravitz J., Stover S.L., Shoureshi R. (2009). Mobile robots in mine rescue and recovery. IEEE Robot. Autom. Mag..

[10] Middleton W., Miller G., Pollman A. Architecture models for coordination of unmanned air and ground vehicles conducting humanitarian assistance and disaster relief. Proceedings of the Conference on Systems Engineering Research.

[11] Kulich M., Kubalik J., Přeučil L. (2019). An Integrated Approach to Goal Selection in Mobile Robot Exploration. Sensors.

[12] Jungyun Bae W.C. (2018). A Heuristic for Path Planning of Multiple Heterogeneous Automated Guided Vehicles. Int. J. Precis. Eng. Manuf..

[13] Vazirani V.V. (2004). Approximation Algorithms.

[14] De Oliveira G.C.R., de Carvalho K.B., Brandão A.S. (2019). A Hybrid Path-Planning Strategy for Mobile Robots with Limited Sensor Capabilities. Sensors.

[15] Tang J., Yang W., Zhu L., Wang D., Feng X. (2017). An Adaptive Clustering Approach Based on Minimum Travel Route Planning for Wireless Sensor Networks with a Mobile Sink. Sensors.

[16] Dahan F., El Hindi K., Mathkour H., AlSalman H. (2019). Dynamic Flying Ant Colony Optimization (DFACO) for Solving the Traveling Salesman Problem. Sensors.

[17] Venkatachalam S., Sundar K., Rathinam S. (2018). A Two-Stage Approach for Routing Multiple Unmanned Aerial Vehicles with Stochastic Fuel Consumption. Sensors.

[18] Bae J., Rathinam S. (2016). A primal-dual approximation algorithm for a two depot heterogeneous traveling salesman problem. Optim. Lett..

[19] Franceschelli M., Rosa D., Seatzu C., Bullo F. (2013). Gossip algorithms for heterogeneous multi-vehicle routing problems. Nonlinear Anal. Hybrid Syst..

[20] Prasad A., Sundaram S., Choi H. Min-Max Tours for Task Allocation to Heterogeneous Agents. Proceedings of the 2018 IEEE Conference on Decision and Control (CDC).

[21] Stodola P. (2018). Using Metaheuristics on the Multi-Depot Vehicle Routing Problem with Modified Optimization Criterion. Algorithms.

[22] Wang X., Golden B., Wasil E. (2015). The min-max multi-depot vehicle routing problem: Heuristics and computational results. J. Oper. Res. Soc..

[23] Sundar K., Rathinam S. (2017). Algorithms for Heterogeneous, Multiple Depot, Multiple Unmanned Vehicle Path Planning Problems. J. Intell. Robot. Syst..

[24] Cho D.H., Jang D.S., Choi H.L. (2019). Sampling-Based Tour Generation of Arbitrarily Oriented Dubins Sensor Platforms. J. Aerosp. Inf. Syst..

[25] Azadeh A., Farrokhi-Asl H. (2019). The close–open mixed multi depot vehicle routing problem considering internal and external fleet of vehicles. Transp. Lett..

[26] Goemans M.X., Williamson D.P., Hochbaum D.S. (1997). The Primal-dual Method for Approximation Algorithms and Its Application to Network Design Problems. Approximation Algorithms for NP-Hard Problems.

[27] Helsgaun K. (2000). An effective implementation of the Lin-Kernighan traveling salesman heuristic. Eur. J. Oper. Res..

[28] Reeds J., Shepp L. (1990). Optimal paths for a car that goes both forwards and backwards. Pac. J. Math..

[29] Dubins L.E. (1957). On Curves of Minimal Length with a Constraint on Average Curvature, and with Prescribed Initial and Terminal Positions and Tangents. Am. J. Math..

[30] Sussmann H.J., Tang G. (1991). Shortest Paths for the Reeds-Shepp Car: A Worked out Example of the Use of Geometric Techniques in Nonlinear Optimal Control.

[31] (2017). IBM ILOG CPLEX Optimization Studio. https://www.ibm.com/us-en/marketplace/ibm-ilog-cplex.

[32] (2009). LKH-2.0.9. http://www.akira.ruc.dk/~keld/research/LKH/.

[33] Bae J. (2014). Algorithms for Multiple Vehicle Routing Problems. Ph.D. Thesis.

